# Bibliometric analysis of tumor marker application in gastric cancer diagnosis from 2019 to 2024

**DOI:** 10.3389/fmed.2025.1547850

**Published:** 2025-05-07

**Authors:** Zhanat Komekbay, Reza Shirazi, Gulnaz Yessultanova, Alisher Garifollin, Anar Tulyayeva, Nurgul Kereyeva, Saule Akhmetova, Abdiraman Kaliev

**Affiliations:** ^1^Department of Histology, West Kazakhstan Marat Ospanov Medical University, Aktobe, Kazakhstan; ^2^Department of Anatomy, School of Biomedical Sciences, Medicine and Health, University of New South Wales, Sydney, NSW, Australia; ^3^Department of Oncology, West Kazakhstan Marat Ospanov Medical University, Aktobe, Kazakhstan; ^4^Department of Pathological Anatomy and Forensic Medicine, West Kazakhstan Marat Ospanov Medical University, Aktobe, Kazakhstan

**Keywords:** tumor marker, gastric cancer, prognosis, immunohistochemistry, diagnosis

## Abstract

Gastric cancer remains a significant global health challenge, ranking fifth worldwide in both incidence and mortality worldwide. Early detection and accurate prognosis are crucial for effective management, yet current diagnostic methods, including tumor markers, often exhibit less sensitivity. This bibliometric analysis investigates trends and key contributions in research on tumor markers for gastric cancer diagnosis from 2019 to 2024. Using Scopus and Web of Science databases, 2,940 articles were analyzed to assess publication trends, prominent authors, institutions, and emerging research themes. Results highlight East Asia, particularly institutions like Fudan University and Nanjing Medical University, as a hub for groundbreaking research. The study identifies key tumor markers and advances in molecular diagnostics, with emphasis on personalized medicine and early detection strategies. Visualization of global collaborations reveals extensive networks driving innovation in this field. While this analysis underscores progress in gastric cancer biomarker research, it also identifies limitations, including language bias and a narrow temporal scope. Future research should prioritize novel biomarkers, integrate advanced technologies like AI, and enhance international cooperation to further improve outcomes for gastric cancer patients.

## 1 Introduction

Stomach cancer is a heterogeneous malignancy associated with environmental and genetic predisposing factors and is the fifth most common cancer worldwide. There were over 968,000 new cases of stomach cancer in 2022 and nearly 660,000 deaths, ranking the disease as fifth in terms of both incidence and mortality worldwide. Thus, according to the Global Cancer Observatory (2022), stomach cancer (4.9%) ranks fifth after lung cancer (12.4%), breast cancer (11.6%), colorectal cancer (9.6%), and prostate cancer (7.3%). Recent indicators have seen a decline in the incidence of stomach cancer, but mortality from this manifestation remains high. For 2022, according to the Global Cancer Observatory, mortality from stomach cancer in the world ranks fifth after lung cancer (18.7%), colorectal cancer (9.3%), and liver cancer (7.8%), female breast (6.9%) (1–7).

Currently, the main challenge in diagnosing stomach cancer is the less sensitivity of the existing methods available for detecting small lesions in the early stages or after radiotherapy and chemotherapy. Moreover, the markers for diagnosing stomach cancer achieved unsatisfactory efficacy. Thus, there is an urgent need to identify new biomarkers to enhance treatment effectiveness in patients with GC (8–10). Today, to predict the clinical course of GC, morphological criteria for the malignancy of the tumor process, such as size, depth of invasion, macroscopic and histological type, are widely used (11, 12). It should be noted that the course of the disease varies significantly within one histological type. In cancer, immunohistochemical (IHC) techniques can predict the clinical course of the disease in different individuals. In this regard, it is necessary to select the most informative markers while also considering complications arising from cancers in other organs (13, 14).

Bibliometric analysis is a quantitative method which applies mathematical and statistical tools to evaluate the inter-relationships and impacts of publications, authors, institutions and countries in a specific research area. Through extracting and analyzing the metrics of each publication including author, institution, country, and keywords, bibliometric analysis is able to determine the development trends or future research directions. Compared with conventional narrative reviews by experts, which often subjectively focus on the progress in a specific research field, bibliometric analysis is advantageous in objectively, comprehensively, and quantitatively summarizing the whole topic based on the best available data (15).

In this study, we conducted a bibliometric analysis and generated visual knowledge maps of relevant publications to analyze the research landscape and trends regarding applying tumor markers for stomach cancer diagnosis from 2019 to 2024, using the Scopus and Web of Science databases. We aim to identify additional promising tumor markers with high sensitivity for this purpose.

## 2 Materials and methods

### 2.1 Eligibility criteria and data source

This bibliometric analysis aimed to analyze the application of tumor markers in stomach cancer diagnosis from 2019 to 2024. The eligibility criteria included original research articles and reviews published in peer-reviewed journals, with only English-language articles considered. Data were collected from major citation databases, **Scopus**, which were chosen due to comprehensive coverage of biomedical research. The search was conducted in November 2024. Metadata for each article were downloaded in BibTeX format from Scopus, and subsequently converted for analysis using the **RStudio** as an excel file (Table 1).

**Table 1:** Codes were used to convert Scopus exported data in RStudio.

library(bibliometrix) library(openxlsx) ## importing scopus dataset scopus_data<−convert2df(“3kw.bib,”dbsource = “scopus,”format = “bibtex”) ##combined both datasets combined<-mergeDbSources(scopus_data, remove.duplicated = TRUE) ##exporting file write.xlsx(combined,"3kw.xlsx”)

### 2.2 Search strategy

**Figure 1:**
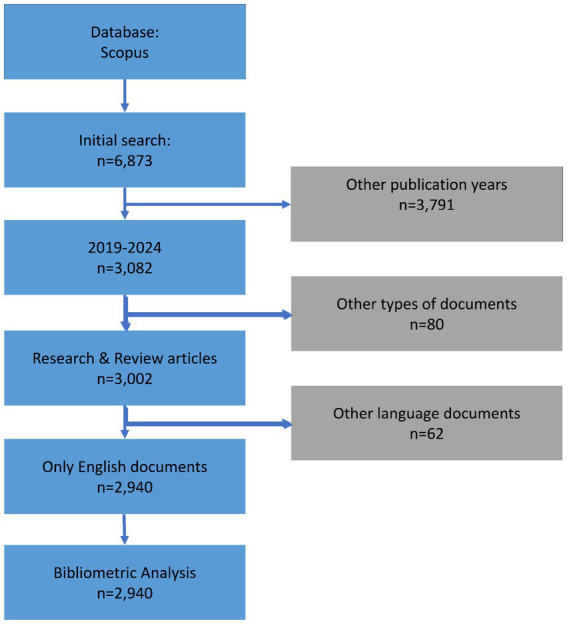
Flowchart depicting the article selection process, from initial retrieval to final inclusion.

The search strategy involved the use of advanced search features in Scopus, with the complete search strategy detailed in Figure 1. Boolean and Wildcard search operators were employed to capture variations in terminology related to tumor markers and gastric cancer. The complete search strategy for databases is outlined in Table 2. Articles deemed irrelevant based on title, abstract, or full-text review were excluded. After obtaining the results, we imported 2,940 articles for bibliometric analyses.

**Table 2:** Scopus database.

Query
(TITLE-ABS-KEY (“Tumor Biomarkers” OR “Tumor Biomarker” OR “Biomarker, Tumor” OR “Biological Tumor Marker” OR “Tumor Marker, Biological” OR “Marker, Biological Tumor” OR “Tumor Markers, Biological” OR “Markers, Tumor Metabolite” OR “Tumor Metabolite Markers” OR “Biochemical Tumor Marker” OR “Tumor Marker, Biochemical” OR “Metabolite Markers, Tumor” OR “Marker, Tumor Metabolite” OR “Metabolite Marker, Tumor” OR “Tumor Metabolite Marker” OR “Tumor Markers, Biologic” OR “Biologic Tumor Markers” OR “Markers, Biologic Tumor” OR “Marker, Biologic Tumor” OR “Biologic Tumor Marker” OR “Tumor Marker, Biologic” OR “Biochemical Tumor Markers” OR “Markers, Biochemical Tumor” OR “Marker, Biochemical Tumor” OR “Tumor Markers, Biochemical” OR “Carcinogen Markers” OR “Markers, Carcinogen” OR “Markers, Neoplasm Metabolite” OR “Neoplasm Metabolite Markers” OR “Marker, Neoplasm Metabolite” OR “Metabolite Marker, Neoplasm” OR “Neoplasm Metabolite Marker” OR “Metabolite Markers, Neoplasm” OR “Biological Tumor Markers” OR “Markers, Biological Tumor” OR “Markers, Tumor” OR “Tumor Markers” OR “Tumor Marker” OR “Marker, Tumor” OR “Cancer Biomarker” OR “Biomarker, Cancer” OR “Biomarkers, Cancer” OR “Cancer Biomarkers”) AND TITLE-ABS-KEY (“Stomach” OR “Stomachs”) AND TITLE-ABS-KEY (“Prognoses” OR “Prognostic Factors” OR “Prognostic Factor” OR “Factor, Prognostic” OR “Factors, Prognostic”)) AND (LIMIT-TO (PUBYEAR, 2019) OR LIMIT-TO (PUBYEAR, 2020) OR LIMIT-TO (PUBYEAR, 2021) OR LIMIT-TO (PUBYEAR, 2022) OR LIMIT-TO (PUBYEAR, 2023) OR LIMIT-TO (PUBYEAR, 2024)) AND (LIMIT-TO (DOCTYPE, “ar”) OR LIMIT-TO (DOCTYPE, “re”)) AND (LIMIT-TO (LANGUAGE, “English”))

### 2.3 Bibliometric analyses

Data management and bibliometric analysis were conducted using the bibliometric package (version 4.3.2) and Biblioshiny, a web-based interface for bibliometric visualization (RStudio 2024.09.0–375, PBC, Boston, MA). The analysis covered a 5-year period for research articles, focusing on publication and citation metrics and citation trends (JOSHI S, 2021, CA CANCER J CLIN). Prolific institutions were identified based on the number of articles related to the applying tumor markers in stomach cancer diagnosis within this timeframe.

This investigation explored interactions among 10 prominent journals, fields of study, and countries contributing to research on the application of tumor markers in stomach cancer diagnosis over the past 5 years. To illustrate global research collaboration, we visualized connections on a world map. A TreeMap visualization depicted the 20 most frequently used keywords in articles published on this topic. Lastly, a “Thematic Map” categorized topics into four domains: primary subjects, specialized subjects, emerging or declining subjects, and overarching foundational subjects.

## 3 Results

Additionally, we created collaborative networks among 606 research journals for research articles using clustering algorithms and data normalization from 2,392 research articles. This process was guided by an association parameter to identify meaningful connections. We identified authors with significant contributions based on the frequency of their published articles. The assessment also included identifying the top 10 most frequently cited documents and leading journals.

### 3.1 Search results

We initially retrieved 6,873 papers from the Scopus database. After applying eligibility criteria and excluding 3,933 ineligible studies, we considered a total of 2,940 papers for this bibliometric analysis (Figure 1).

### 3.2 Key attributes of the included studies

Among the 2,940 articles, a majority of the studies were original research articles (81.4%), with the remainder comprising reviews (18.6%). The most common research areas included molecular biology, clinical oncology, and pathomorphology, reflecting the interdisciplinary nature of tumor marker research in gastric cancer diagnosis.

### 3.3 Trend of publication and citation

**Figure 2:**
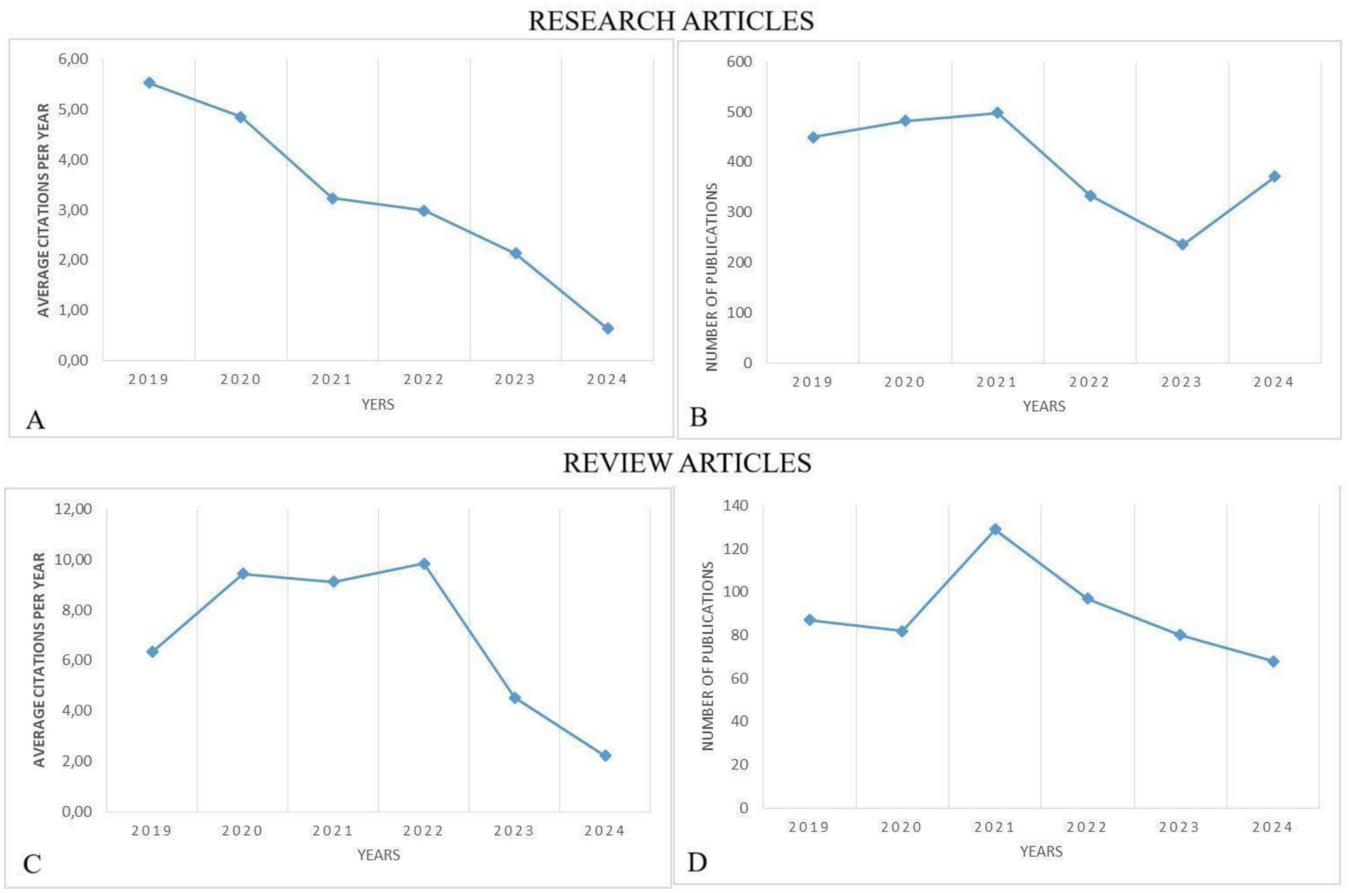
Average citations in **(A)** research study publications and **(B)** global annual trends, as well as **(C)** review article citations and **(D)** publications, biomarkers and GC research from 2019 to 2024.

Figure 2 presents the trends in average citations and publication counts for research and review articles related to gastric cancer and biomarkers from 2019 to 2024. Graph A illustrates the average annual citations of research articles over this period, showing a consistent decline, with citations steadily decreasing each year. Graph B displays the global number of research article publications, which, despite some fluctuations, remains relatively stable, with a slight decrease in recent years. Graph C highlights the average annual citations of review articles, revealing an upward trend from 2019 to 2022, followed by a sharp decline thereafter. Graph D shows the number of published review articles, peaking around 2022 before steadily dropping.

The average citations for both research and review articles on gastric cancer and biomarkers have declined in recent years, with review articles initially experiencing growth before decreasing. The number of publications shows slight variations, potentially reflecting shifts in research focus or citation patterns.

### 3.4 Most relevant affiliations

In the context of research articles focused on the investigation of the applying tumor markers in stomach cancer, an assessment of institutional productivity reveals Fudan University as the leading institution in terms of output. This institution has made significant contributions to the field by presenting a total of 106 research papers on the subject (Table 3). Other top institutions included Nanjing Medical University and Sun Yat-Sen University, indicating a strong concentration of research output in East Asia.

**Table 3:** The top 10 affiliations that published research and review articles on biomarkers and GC research from 2019 to 2024.

Affiliation	Articles
**Research articles**	
Fudan University	106
Nanjing Medical University	85
Sun Yat-Sen University	79
Peking University Cancer Hospital and Institute	71
Central South University	67
Huazhong University of Science and Technology	66
Southern medical university	59
Sun Yat-Sen University Cancer Center	59
Shandong University	56
Affiliated Hospital of Nantong University	55
**Review articles**	
Mashhad University of Medical Sciences	36
Shahid Beheshti University of Medical Sciences	36
Sichuan University	22
Tabriz University of Medical Sciences	22
Central South University	20
Shejiang University	18
Lanzhou University	17
Islamic Azad University	16
Huazhong University of Science and Technology	11
Southwest Medical University	11

Table 3 offers an evaluation of institutional productivity within research on the application of tumor markers in stomach cancer. It identifies Mashhad University of Medical Sciences as a primary contributor, with a total output of 36 review articles. Additional prominent institutions include Shahid Beheshti University of Medical Sciences and Tabriz University of Medical Sciences, suggesting a significant concentration of research activity in this field in Iran. This distribution underscores the role of these institutions in advancing knowledge on tumor markers in stomach cancer research.

### 3.5 Most contributing authors and their collaboration network

**Figure 3:**
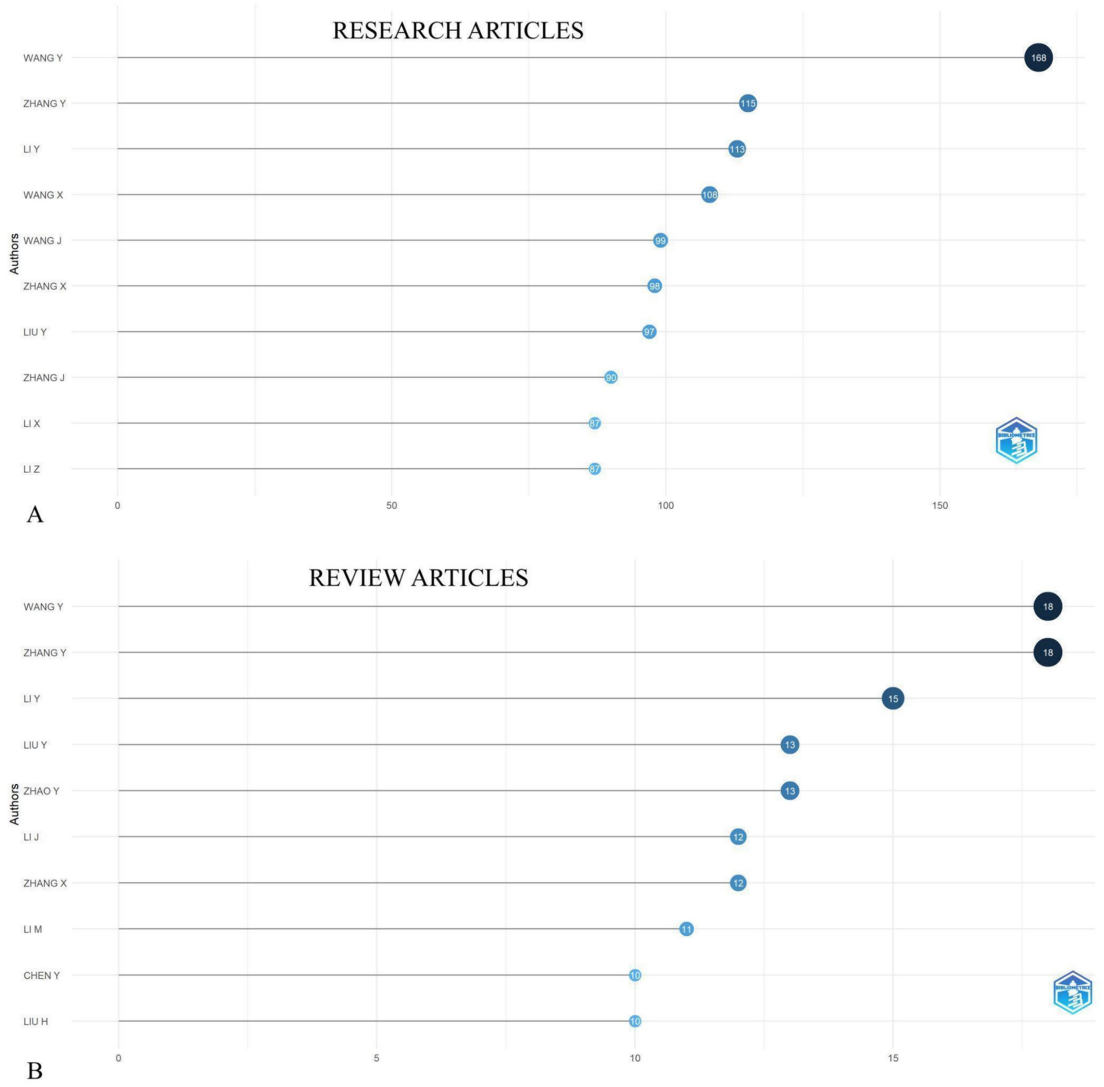
Top 10 most prolific authors of research articles **(A)** and review articles **(B)** in the field of biomarkers and GC research from 2019 to 2024.

Leading authors such as **Wang Y** and **Zhang Y** had the highest number of published research articles on tumor markers in gastric cancer, with **168** and **115** articles and review articles for both 18, respectively (Figures 3A,B). Their collaboration networks were analyzed, revealing key partnerships with researchers across Europe and North America.

**Figure 4:**
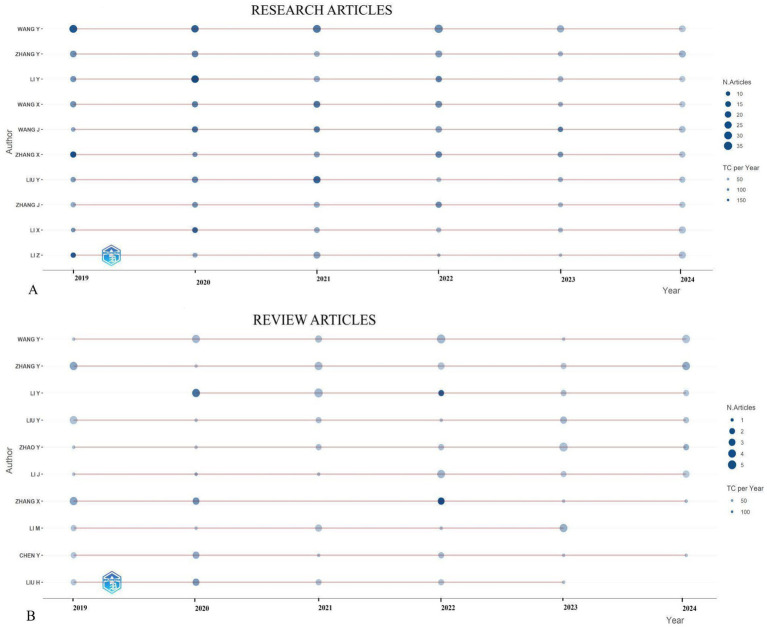
Top 10 most prolific authors’ research article **(A)** and review article **(B)** production over time (2019–2024) regarding biomarkers and GC research.

Figure 4 presents a graphical representation of the top 10 authors over time, spotlighting the most prolific contributors in the field. These prolific authors are those who have made substantial contributions to the field, as determined by their h-index, indicating that each author has amassed at least “h” citations for their published papers. In Figure 4, the size of the circles corresponds to the number of articles authored, with larger circles denoting a higher volume of publications. Furthermore, the color of the circles represents the total citations per year, where a darker shade indicates a greater number of citations.

### 3.6 Most productive journals

**Figure 5:**
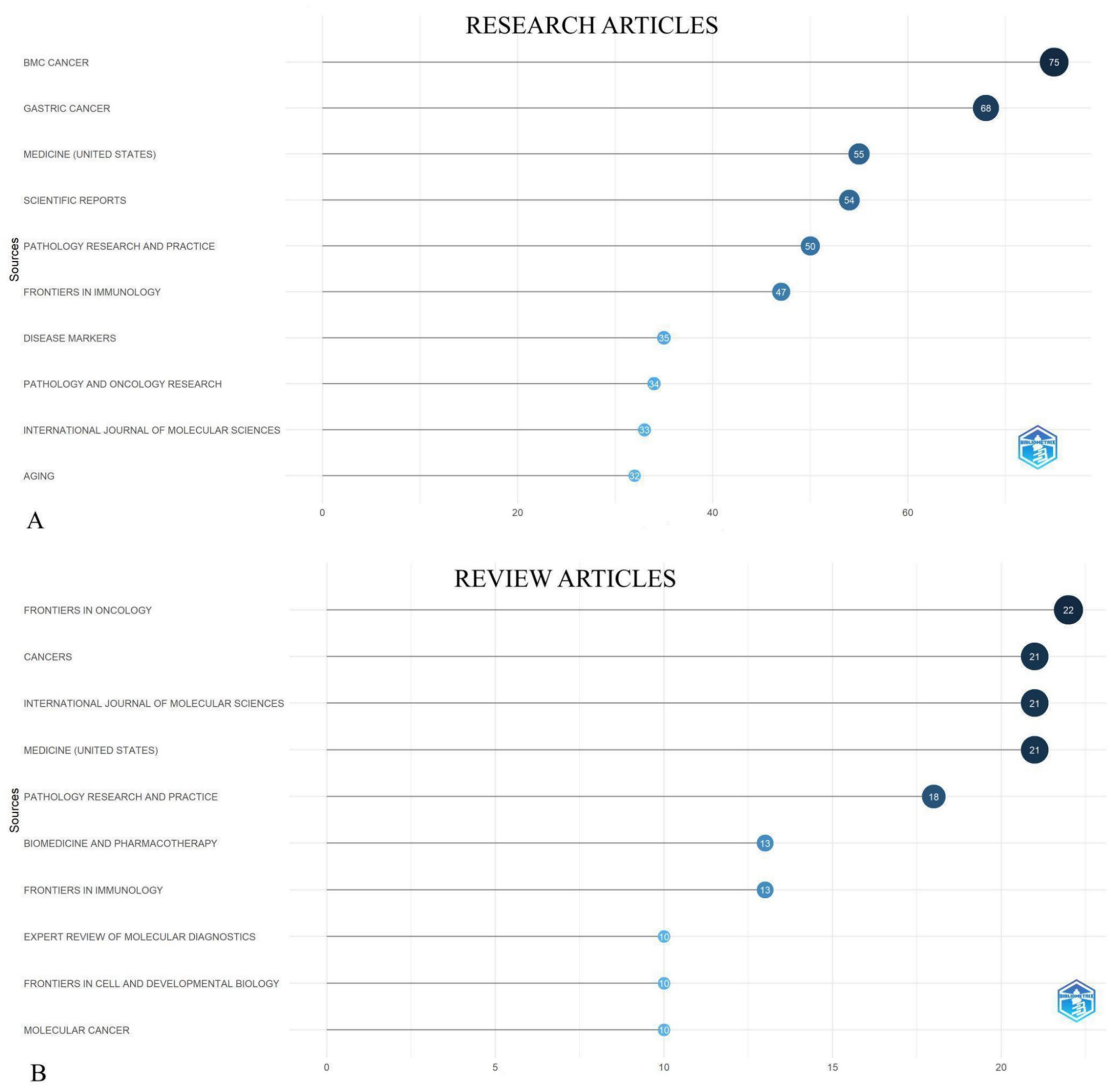
The top 10 most productive journals on biomarkers and GC in research articles **(A)** and review articles **(B)**, respectively from 2019 to 2024.

Among the published research articles in our field of interest, 75 research articles were featured in the journal “BMC Cancer” followed by 68, 55, 54, 50 research articles in the journals “Gastric Cancer,” “Medicine (United States),” “Scientific Reports,” “Pathology Research and Practice” respectively as shown in Figure 5A.

**Figure 6:**
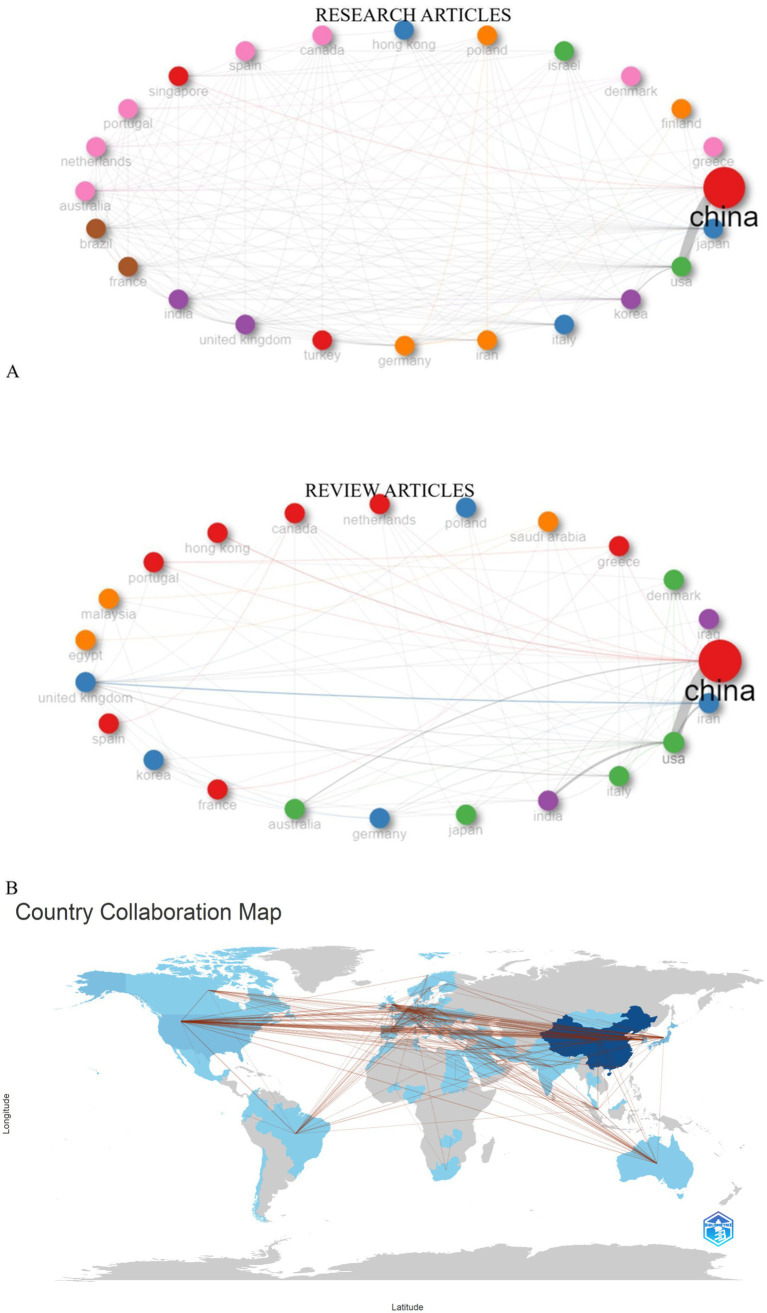
A visual representation of countries’ collaborative networks in the publication of research articles **(A)** and review **(B)** focusing on the application of the tumor markers in stomach cancer diagnosis from 2019 to 2024. The map highlights collaborative efforts between various countries, illustrating the global nature of research in this field.

Most productive journals between 2019 and 2024, as shown in Figure 5B were “Frontiers in oncology” with 22 articles, “Cancers,” “International Journal of Molecular Sciences,” “Medicine (United States)” each with 21 articles and “Pathology Research and Practice”with 18 review articles published on GC and biomarkers field. The network diagram of international collaboration in publications on the studied topic shows that China holds a leading position in terms of both the number of publications and the degree of collaboration. Other active contributors include the United States, the United Kingdom, Germany, and Australia (Figure 6).

### 3.7 World research production and collaborations

**Figure 7:**
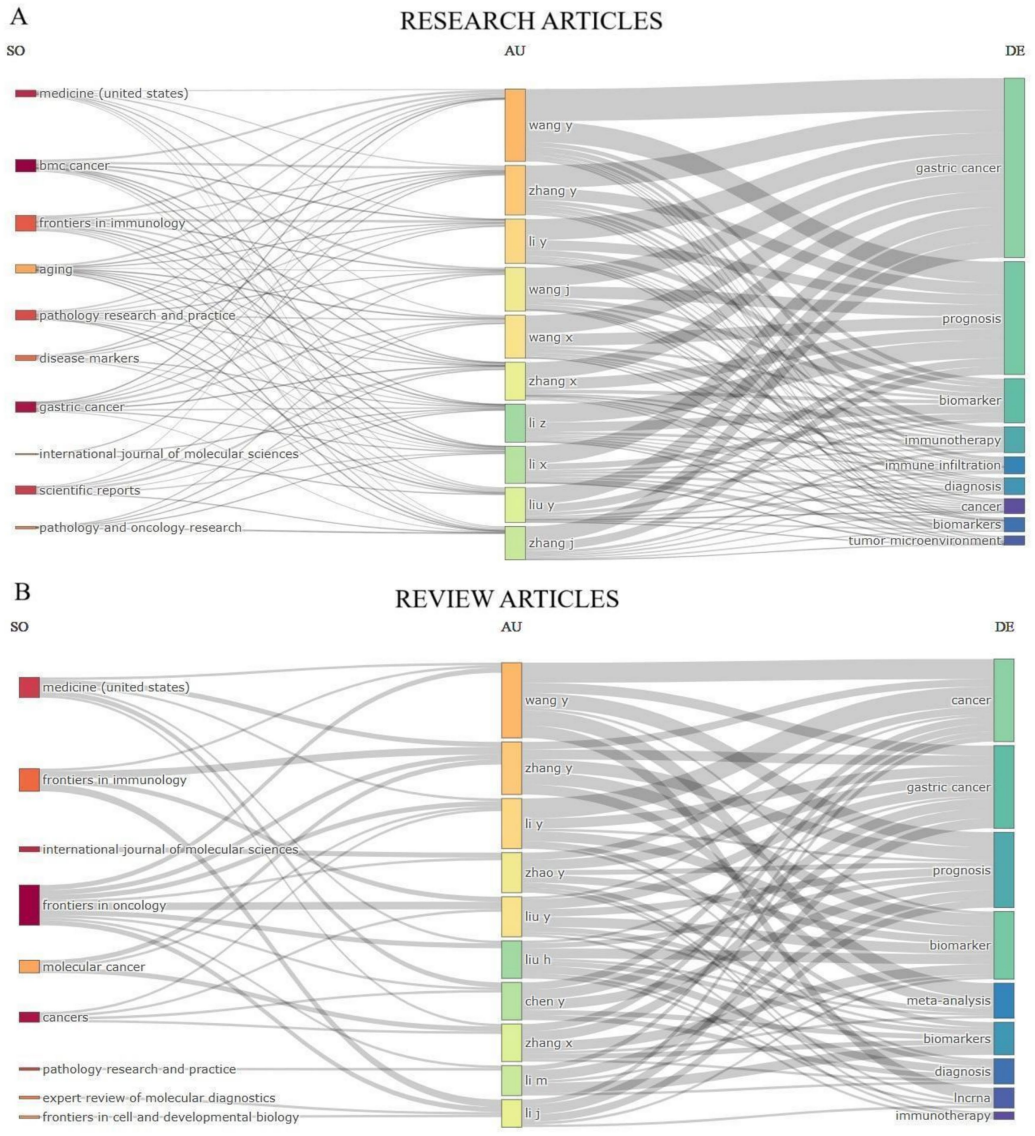
Three-Fields Plot that illustrates the interconnections between the top 10 journals, authors, and keywords that have made contributions to research articles **(A)** and review articles **(B)** on the application of the tumor markers in stomach cancer diagnosis. This plot visualizes the incoming and outgoing flows of influence among these key elements in the research field.

To depict the contributions from the top 10 journals, authors, and keywords in exploring the applying tumor markers in stomach cancer diagnosis, we have created a graphical representation that illustrates the interactions within these domains. This depiction illustrates the inbound and outbound interactions among these respective domains in Figure 7.

This visualization effectively maps the collaboration between scientific output, key contributors, and focal research topics, providing insights into influential authors and trending areas within cancer research.

### 3.8 TreeMap and thematic map

**Figure 8:**
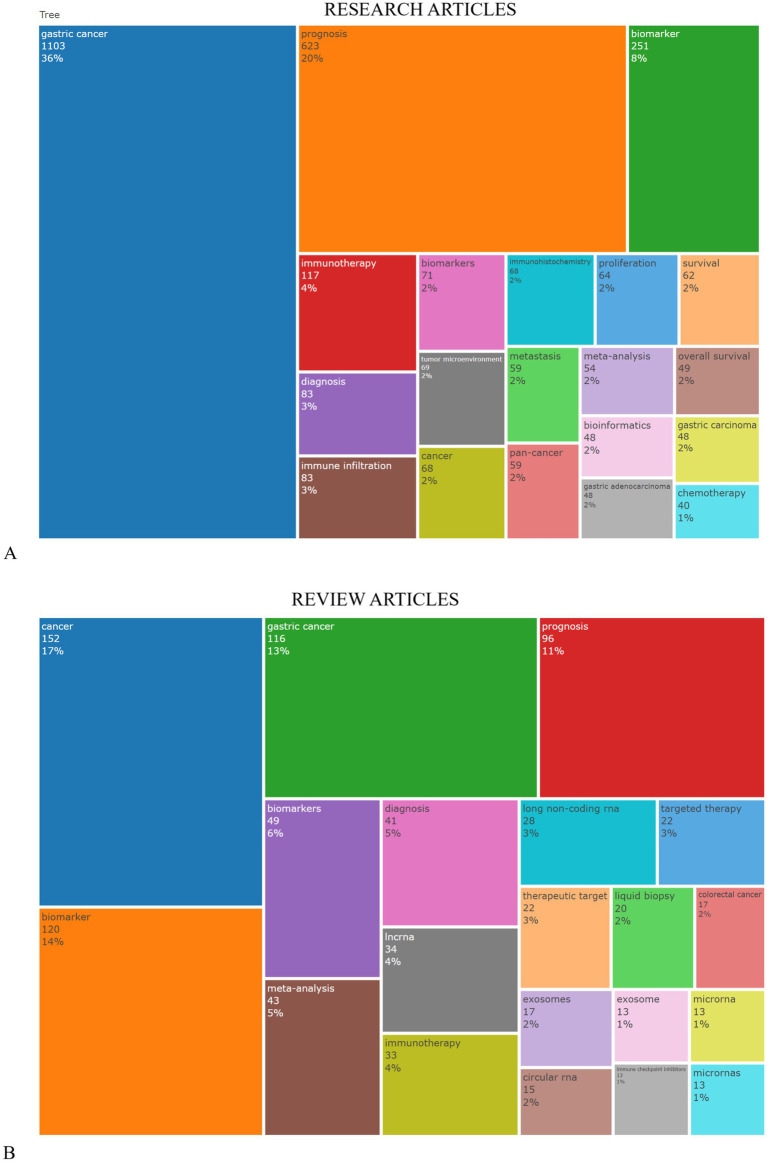
TreeMap visualization that highlights the top 20 author’s keywords commonly found in research articles related to tumor markers in stomach cancer. **(A)** refers to data from original research articles, while part **(B)** presents data from review articles, with an emphasis on the use of keywords related to the diagnosis of gastric cancer.

Figure 8A displays a TreeMap visualization that highlights the top 20 keywords frequently used by authors in research articles related to applying tumor markers in stomach cancer diagnosis. It is noteworthy that three keywords, namely, “gastric cancer” (constituting 36% of usage), “prognosis” (comprising 20% of usage), and “biomarker” (comprising 8% of usage), prominently recur within this research domain. While Figure 8B presents a showcasing the top 20 keywords in review articles notably, three keywords including “cancer” (17%), “gastric cancer” (making up 13% of usage), and “biomarker” (representing 14% of usage) stand out as recurring themes in this field.

### 3.9 Overview of gastric cancer literature trends (2019–2024)

**Figure 9:**
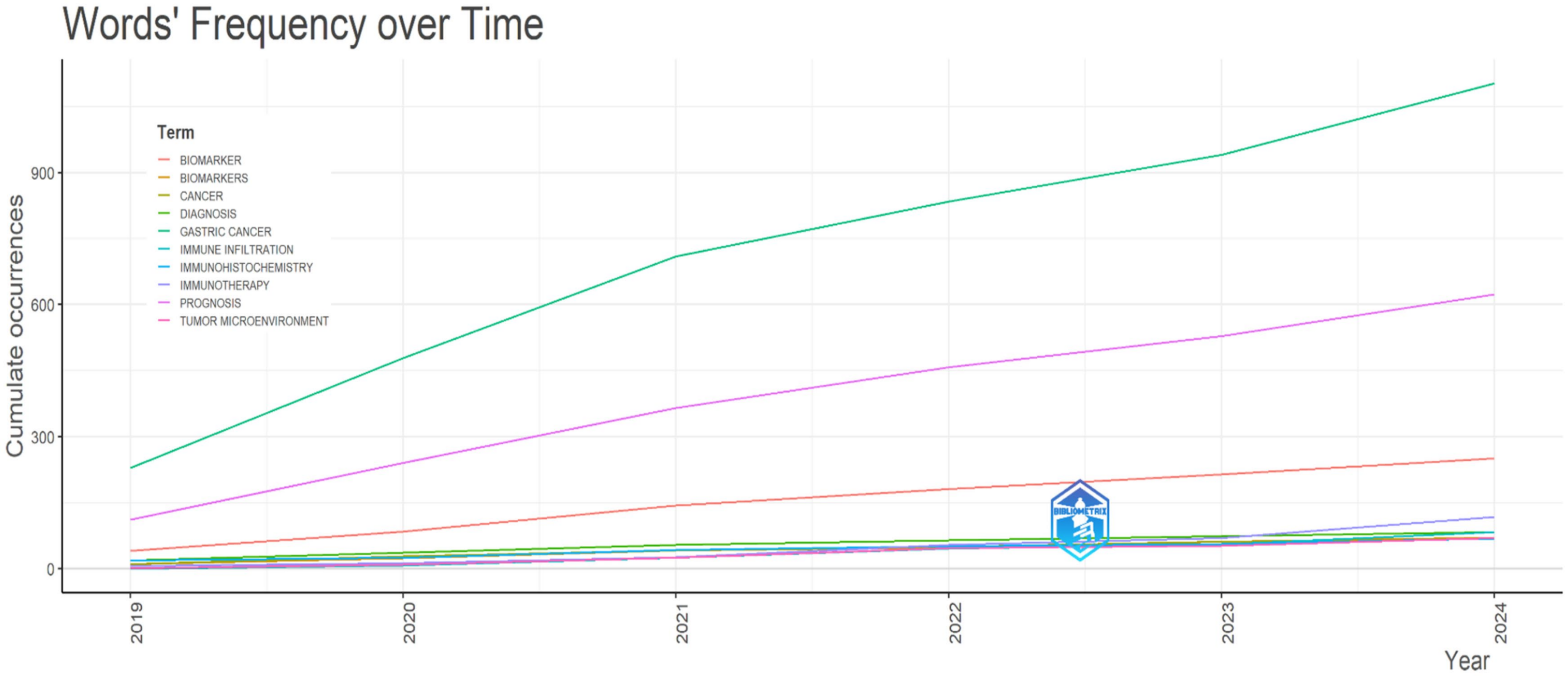
Trends and emerging themes in gastric cancer research studies (2019–2024). This figure highlights the evolving attention of researchers and clinicians toward prognosis within the context of GC studies during the period 2019–2024.

For a focused examination of GC literature in recent years, we conducted an in-depth analysis of publications spanning the period 2019 to 2024. Our investigation revealed a notable surge in research interest and publications related to GC during the specified timeframe (Figure 9).

## 4 Discussion

The current bibliometric analysis provides a comprehensive overview of research trends in tumor marker applications for gastric cancer (GC) diagnosis from 2019 to 2024, highlighting significant advancements in molecular diagnostics, institutional contributions, and emerging research themes that shape the field. The increasing number of publications in the recent years underscores the growing interest of tumor biomarkers and their potential to transform early detection and personalized treatment strategies.

### 4.1 Global research trends

The analysis reveals an increased trend in publications on GC biomarkers, with 81.4% original research articles and 18.6% reviews, confirming heightened research activity driven by the high global burden of GC and advancements in molecular diagnostics (16–21). Moreover, a prominent portion of the research is contributed from East Asia including China, Japan and South Korea due to their high GC incidence rate (1, 2). Institutions such as Fudan University, Nanjing Medical University, and Sun Yat-Sen University lead in publication volume, contributing pioneering work in molecular biology and clinical oncology (22–26). North America and Europe also play significant roles, fostering robust international contribution that enhance knowledge exchange and innovation (27–31). These global networks, visualized in collaborative maps, underscore the interdisciplinary and transnational nature of GC biomarker research and advancements in the identification and validation of novel tumor markers (16–21).

### 4.2 Institutional and author contributions

Fudan University, Nanjing Medical University, and Sun Yat-Sen University remain at the forefront, driving innovation in the field of GC biomarkers. Their vast contributions include pioneering works in the fields of molecular diagnostics and clinical oncology, which have supported the advancement of methods for early detection and treatment approaches (22–26).

Among these are highly cited authors such as Wang Y and Zhang Y. Their extensive collaboration networks, spanning Asia, Europe, and North America, highlight the importance of interdisciplinary partnerships in advancing GC research. The increasing prevalence of multi-center studies and cross-institutional collaborations further illustrates the value of shared expertise and resources in driving scientific progress (27–31).

### 4.3 Key research themes and emerging trends

The bibliometric mapping of keywords, authors, and journals reveals main and emerging themes in GC biomarker research, with gastric cancer (36%), prognosis (20%), and biomarker (8%) as the most frequent keywords. These themes emphasize the critical role of tumor biomarkers in predicting disease progression and guiding personalized treatment strategies. Genetic and epigenetic markers, such as HER2, microsatellite instability (MSI), and circulating tumor DNA (ctDNA) play crucial roles in diagnosis and prognosis (22). For instance, HER2-targeted therapies have shown promise in improving outcomes for HER2-positive GC patients, though challenges like heterogeneity in expression persist (19, 32).

Emerging trends include the integration of novel technologies to enhance biomarker sensitivity and specificity. Nanotechnology, for example, is being explored for early detection of small lesions, addressing the limitations of traditional diagnostic methods (8). Additionally, the application of artificial intelligence (AI) and machine learning is gaining traction, with models being developed to analyze large-scale biomarker data and refine diagnostic algorithms (24). These innovations enable automated identification of tumor markers, improving diagnostic efficiency and reducing observer bias (33).

Immunohistochemistry (IHC) techniques continue to be widely used for improving diagnostic specificity, with recent advances focusing on novel markers like Claudin-18 and Ki-67 to enhance prognostic accuracy across histological subtypes (9, 13, 14). The integration of IHC with digital pathology and imaging-based analysis is enhancing precision in GC subtype differentiation (31).

### 4.4 Productive journals and collaborative networks

The most impactful articles have been published within prestigious journals like the BMC Cancer, Gastric Cancer, and Scientific Reports, serving as an important source for the diffusion of research output. The growing number of international research collaborations further strengthens knowledge exchange, fostering innovation and accelerating the translation of scientific discoveries into clinical practice. Finally, the collaborative networks, as depicted by the bibliometric maps, are very significant in terms of institutions and countries’ partnerships for knowledge flow and creation (32–37).

### 4.5 Strong and weak points of the analysis

This bibliometric analysis provides an extensive quantitative overview of the field, taking into consideration influential researchers, institutions, and tendencies.

However, certain limitations should be acknowledged. The limitations the exclusive focus on English-language publications and the Scopus database, which may exclude significant non-English studies or those indexed elsewhere. The five-year temporal scope, while focused, may miss longer-term trends or nascent fields. Despite these constraints, this study provides critical insights into the current landscape of GC biomarker research, highlighting key advancements and areas for further exploration.

### 4.6 Clinical and research implications

The findings emphasize the potential of tumor markers to revolutionize gastric cancer management. Clinically, novel biomarkers promise improved early detection, addressing the challenge of identifying small lesions at treatable stages (8, 12). Personalized Treatment Strategies, driven by molecular insights, are poised to enhance therapeutic efficacy, particularly through targeted therapies and immunotherapies (22–26). From a research perspective, strengthening global collaborations can accelerate innovation and address disparities in research funding and access to advanced diagnostics.

## 5 Conclusion

This bibliometric analysis demonstrates the significant progress made in the application of tumor markers for gastric cancer diagnosis between 2019 and 2024. Key institutions and researchers are driving innovation, and the global collaborative networks revealed through this analysis highlight the importance of international cooperation. Future research should continue to explore novel biomarkers and diagnostic techniques, with the aim of improving early detection and personalized treatment strategies for gastric cancer.

## Data Availability

The datasets presented in this article are not readily available because this bibliometric analysis aimed to analyze the application of tumor markers in stomach cancer diagnosis from 2019 to 2024. The eligibility criteria included original research articles and reviews published in peer-reviewed journals, with only English-language articles considered. Data were collected from major citation databases, Scopus, which were chosen due to comprehensive coverage of biomedical research. The search was conducted in November 2024. Requests to access the datasets should be directed to Zhanat Komekbay, Zhanat.ru@inbox.ru.
